# Assessing the Efficacy of Artificial Intelligence Platforms in Answering Dental Caries Multiple-Choice Questions: A Comparative Study of ChatGPT and Google Gemini Language Models

**DOI:** 10.3390/dj14020072

**Published:** 2026-01-27

**Authors:** Amr Ahmed Azhari, Walaa Magdy Ahmed, Abdulaziz Alhamadani, Amal Alfaraj, Min Zhang, Chang-Tien Lu

**Affiliations:** 1 Department of Restorative Dentistry, Faculty of Dentistry, King Abdulaziz University, Jeddah P.O. Box 80200, Saudi Arabia; aaaazhari@kau.edu.sa; 2 Department of Data Science and Business Analytics, Florida Polytechnic University, Lakeland, FL 33801, USA; aalhamadani@floridapoly.edu; 3 Department of Prosthodontics, College of Dentistry, King Faisal University, Al Aahsa P.O. Box 80209, Saudi Arabia; asalfaraj@kfu.edu.sa; 4 Department of Computer Science, Virginia Tech, Northern Virginia Center, Falls Church, VA 22043, USA; minzhang23@vt.edu (M.Z.); ctlu@vt.edu (C.-T.L.)

**Keywords:** large language models, ChatGPT, Gemini, dental education, dental caries, multiple-choice questions, simulation, artificial intelligence

## Abstract

**Objective**: This study aimed to compare the accuracy of two large language models (LLMs)—ChatGPT (version 3.5) and Google Gemini (formerly Bard)—in answering dental caries-related multiple-choice questions (MCQs) using a simulated student examination framework across seven examination lengths. **Materials and Methods**: A total of 125 validated dental caries MCQs were extracted from Dental Decks and Oxford University Press question banks. Seven examination groups were constructed with varying question counts (25, 35, 45, 55, 65, 75, and 85 questions). For each group, 100 simulations were generated per LLM (ChatGPT and Gemini), resulting in 1400 simulated examinations. Each simulated student received a unique randomized subset of questions. MCQs were answered by each LLM using a standardized prompt to minimize ambiguity. Outcomes included mean score, passing rate (≥60%), and performance differences between LLMs. Statistical analyses included independent *t*-tests, one-way ANOVA within each LLM, and two-way ANOVA examining interactions between LLM type and question count. **Results**: Across all seven examination formats, Gemini significantly outperformed ChatGPT (*p* < 0.001). Gemini achieved higher passing rates and higher mean scores in every examination length. One-way ANOVA revealed significant score variation with increasing exam length for both LLMs (*p* < 0.05). Two-way ANOVA demonstrated significant main effects of LLM type and question count, with no significant interaction. Randomization had no measurable effect on Gemini performance but influenced ChatGPT scores. **Conclusions**: Gemini demonstrated superior accuracy and higher passing rates compared to ChatGPT in all simulated examination formats. While both LLMs struggled with complex caries-related content, Gemini provided more reliable performance across question quantities. Educators should exercise caution in relying on LLMs for automated assessment or self-study, and future research should evaluate human–AI hybrid models and LLM performance across broader dental domains.

## 1. Introduction

In recent years, artificial intelligence (AI) integration into healthcare has surged, aiming to improve patient outcomes, reduce treatment costs, and enhance care [1]. AI has proven valuable in image processing, enabling radiologists to make timely diagnoses of abnormalities and potential health risks [2,3,4,5,6,7]. AI algorithms can analyze vast amounts of patient data stored in electronic health records to identify patterns indicative of health issues. This expedites the review process, which is time-intensive for healthcare professionals. Another significant contribution of AI in healthcare is drug discovery, which aids the development of new treatments [8]. AI-powered virtual assistants grant patients greater agency in managing their healthcare by offering personalized advice based on data analysis [9]. Predictive analysis is another promising application of AI for assisting doctors in preventing future health issues [10,11,12].

AI has the potential to revolutionize education, making it more personalized and efficient. AI algorithms can analyze student data to tailor learning experiences to individual strengths, weaknesses, and learning styles [13]. This data-driven approach allows for early identification of students at risk of failure, enabling timely intervention and support. China has implemented this technique [14]. China’s new initiative to integrate AI into its educational system uses AI-powered personalized learning platforms that adapt to the learning patterns and tempo of individual students. The objective is to increase student engagement and learning outcomes while simultaneously preparing the next generation of AI experts. This initiative may have significant implications for the future of education worldwide, as other nations may follow China’s lead in incorporating AI into their educational systems. AI can also provide personalized tutoring services, aiding students in their studies and assignments [15]. Grading and assessment tasks can be automated using AI, reducing teachers’ workload and ensuring more consistent and objective evaluations [16]. Additionally, AI can generate educational content, such as quizzes and study guides, adapting the difficulty level based on students’ responses [17,18]. Natural language processing facilitates interactive support for students by answering questions and engaging in natural language interactions [19].

The recent development of large language models (LLMs) within AI has presented new opportunities for healthcare and education. AI and LLMs have rapidly expanded into educational and clinical environments, including dentistry [2,3,4,17]. LLMs such as ChatGPT (OpenAI) [20] and Google Gemini (formerly Google Bard, rebranded in 2024 as Google Gemini) are increasingly used by students for information retrieval, examination preparation [17], and clinical reasoning support [21]. Their ability to generate natural-language answers offers potential value in dental education; These LLMs are trained using reinforcement and supervised learning methods with big data, allowing them to generate novel sequences of words based on human language patterns [22,23]. The objective of LLMs is to create a potent, adaptable natural language processing instrument capable of comprehending, generating, and manipulating language resembling that of humans, with exceptional quality and precision [24]. However, their accuracy, reliability, and consistency remain insufficiently understood, particularly within specialized domains such as cariology.

Large language models (LLMs) are advanced artificial intelligence systems trained on massive text datasets using deep learning to understand, generate, and predict human-like language [25]. These models rely on the Transformer architecture, which uses self-attention mechanisms to process entire sequences efficiently and capture long-range linguistic patterns. LLMs are typically developed through pre-training, where they learn general language structures from vast datasets from the internet (books, articles, websites), followed by fine-tuning, where human feedback or domain-specific data refine their performance for targeted tasks. Variations in model size, training data, and fine-tuning approaches contribute to differences in capability between systems such as ChatGPT and Google Gemini. Notably, Gemini, introduced as a lightweight evolution of language models that was designed for dialogue applications (LaMDA) with an emphasis on reducing bias and improving safety.

In education, LLMs have demonstrated potential for analyzing student-generated text, identifying performance patterns, and supporting personalized learning [26]. They can facilitate instructional design, provide automated feedback, and assist educators in monitoring student needs [27]. LLMs can also evaluate written assignments based on clarity, coherence, and domain-appropriate terminology [28]. Their conversational capabilities enable virtual assistants and tutoring agents that support independent learning and real-time guidance [29,30].

In health professions education, LLMs show promise for reinforcing foundational knowledge and supporting examination preparation. ChatGPT’s strong performance on USMLE-style questions demonstrates its ability to interpret medical terminology and reasoning patterns [31], with similar applications reported in medical, pharmacological, and dental education [32]. Within dentistry, LLMs such as ChatGPT and Gemini can provide accessible information on the prevention, diagnosis, and management of dental caries, offering general guidance on oral hygiene and treatment options [33]. For clinicians, LLMs may support treatment planning, enhance patient communication, and assist with follow-up reminders [34], suggesting growing relevance to dental practice and education.

Despite these advantages, LLM-generated content is not always reliable. Responses may be inaccurate, incomplete, or outdated, particularly for rapidly evolving clinical information. Studies have reported errors and outdated recommendations in dental content generated by ChatGPT after 2021 [21]. These limitations highlight the need for critical evaluation, human oversight, and ethical consideration, especially regarding bias and misinformation when integrating LLMs into dental and medical educational contexts.

Dental caries, a widespread chronic bacterial infection that causes the demineralization of tooth structures, requires early detection and treatment to prevent disease progression and tooth loss [35]. Also, dental caries remains a fundamental topic in dental curricula. AI, particularly machine, and deep learning, has been explored for dental caries detection at different stages [36,37,38,39,40,41,42,43,44]. Research on LLM in the field of dental caries is important but has not yet been explored. While LLMs have achieved breakthroughs in natural language understanding, whether they can solve domain-specific knowledge like domain experts remains unknown. Additionally, as LLMs are being widely used by students, the reliability of LLM results is crucial. The assessment of LLMs’ ability in caries can provide better guidance to caries-related students and teachers when using LLMs. Therefore, investigating whether an LLM possesses knowledge related to dental caries is highly necessary. Although no specific studies on ChatGPT and/or Google Bard (rebranded in 2024 as Google Gemini) and dental caries have been conducted, LLMs, including ChatGPT, have been used in dental and healthcare research to analyze large-scale datasets from various sources [33]. Although the explicit application of ChatGPT in dental caries diagnosis and management has not been studied, its potential for analyzing and processing natural language data holds promise for dental and healthcare research. Therefore, the objective of this study was to compare the performance of ChatGPT and Gemini across seven examination lengths using a simulated student model. We hypothesized that Gemini would demonstrate higher accuracy and higher passing rates than ChatGPT across all examination formats.

## 2. Materials & Methods

Ethical Approval: Ethical approval for this study was granted by the Faculty of Dentistry, King Abdulaziz University, Jeddah, Saudi Arabia (112-06-23). Approval Date: 16 November 2023. Data collection and all experimental procedures were conducted in May 2023.

Study Design: This observational, cross-sectional simulation study aimed to assess the ability of two large language models (LLMs)—ChatGPT and Gemini—to answer multiple-choice questions (MCQs) related to dental caries. A total of 1400 simulated students were generated, with each model completing 700 examinations drawn from validated cariology question banks. Because the study relied on simulated students rather than human participants, a traditional a priori human sample-size calculation was not applicable. Instead, 100 Monte Carlo repetitions were generated for each of the seven examination lengths for each LLM. This produced 1400 simulated exams and ensured more than 0.99 statistical power to detect performance differences of 5% or greater.

Question Bank Development: The selection of two different databases was to preserve full content coverage across caries-related subdomains (diagnosis, risk assessment, prevention, and management). An expert review was conducted to identify any gaps in topic representation. All added questions met the validation criteria regarding clarity, discrimination index, and relevance to the competency framework. The question bank comprised a total of 125 validated MCQs covering multiple dimensions of dental caries. A total of 115 items were sourced from the Dental Decks repository, a widely used educational resource that includes validated questions across multiple dental disciplines, including microbiology, pathology, restorative dentistry, preventive dentistry, and oral surgery. During expert review, minor gaps were identified in three subdomains of cariology: caries prevention, risk assessment, and early lesion diagnosis. To address these gaps and ensure comprehensive content coverage, 10 additional validated MCQs were incorporated from the Oxford University Press (OUP) online question resource https://global.oup.com/uk/orc/health/geissler13e/student/mcqs/ch26/, accessed on 3 November 2023.

Two cariology content experts independently reviewed all MCQs for accuracy, clarity, relevance, and alignment with learning objectives. Items were revised based on consensus feedback to ensure clarity, avoid bias, and maintain appropriate difficulty. Because all questions were already validated prior to inclusion, no further prioritization was required. An example MCQ used in the study was: “Which of the following bacteria is most associated with dental caries development?” with answer choices Actinomyces naeslundii, Streptococcus mutans, Lactobacillus acidophilus, and Veillonella parvula.

**Figure 1:**
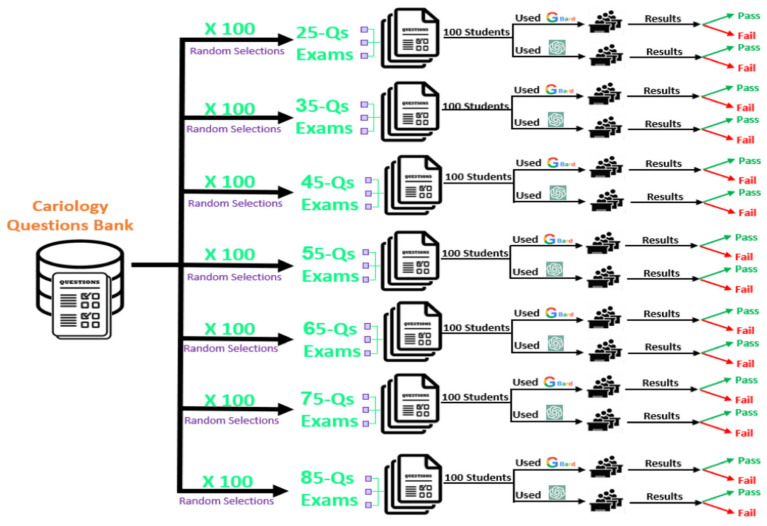
Study workflow for simulated examination generation. A schematic representation of the study design, including MCQ validation, randomization of question sets, simulated student creation, LLM querying (ChatGPT and Gemini), and scoring procedures.

Construction of Examination Sets: Seven examination formats were developed to simulate different test lengths, consisting of 25, 35, 45, 55, 65, 75, and 85 questions. For each examination length, 100 unique examinations were generated using Python’s random.sample() function to select questions without replacement, resulting in 700 distinct examinations across all groups. Each group of examinations was then assigned to 200 simulated students, with 100 students evaluated using Gemini and 100 using ChatGPT. Each student received a unique set of questions that was not duplicated within the group. In total, 1400 simulated examination attempts were created. All simulations were executed using Python (version 3.11) by the study’s data scientist, and the outputs were independently reviewed by the senior author for accuracy and reproducibility. Figure 1 provides an overview of the workflow.

Simulated Student Framework: A total of 1400 simulated student attempts were generated, with 700 examinations answered using ChatGPT and 700 using Gemini. Each simulated student received a distinct MCQ combination. This Monte Carlo simulation framework was designed to approximate the natural variation encountered in real student cohorts while preserving controlled, repeatable testing conditions for both models.

LLM Prompting Protocol and Safeguards: Each MCQ was submitted individually to each LLM using a standardized prompt to avoid contextual contamination. The prompt stated: “You are answering a single multiple-choice question from dentistry. Choose only one best answer from A, B, C, or D. Return only the letter.” No prior conversation history, contextual information, or memory carryover was permitted. Explanations were disabled to reduce response variability. Each question was therefore evaluated independently under strictly controlled and identical conditions for both LLMs.

Scoring Procedure: Each LLM’s response (A–D) was matched against the validated answer key. Raw scores, percentage scores, and pass/fail outcomes were calculated for each examination, with a passing criterion defined as achieving a minimum of 60% correct responses. Mean scores and standard deviations were computed for each examination group and for each model.

Statistical Analysis: All statistical analyses were conducted using Python tools (NumPy, SciPy, and Pandas libraries) for data handling, computation, and statistical testing. Descriptive statistics were calculated to determine mean scores and passing rates across all examination groups. Independent-sample *t*-tests were performed to compare ChatGPT and Gemini performance for each exam length. To assess whether examination length influenced performance within each model, a seven-group one-way ANOVA was conducted separately for ChatGPT and Gemini. A two-way ANOVA was then used to evaluate the main effects of LLM type (ChatGPT vs. Gemini), examination length, and their interaction. Statistical significance for all analyses was set at α = 0.05.

## 3. Results

Table 1 presents the mean examination scores and standard deviations for both LLMs across all seven examination lengths. Gemini consistently produced higher mean scores than ChatGPT. Across all examination groups, ChatGPT mean scores ranged from 51.0% to 53.2% (Table 1), whereas Gemini mean scores ranged from 60.4% to 61.6%. Passing rates (≥60%) also differed markedly between models. Gemini passing rates ranged from 49% to 59%, depending on exam length, while ChatGPT passing rates ranged from 4% to 14%, indicating substantially lower accuracy (Table 2). These descriptive results suggest Gemini outperformed ChatGPT regardless of exam length.

The passing rates of the students are shown in Table 2. In all seven groups, 50–59% of students who used Gemini passed, whereas only 4–14% of students who used ChatGPT passed. For Gemini, the highest passing rate was 59% in the 25-question examination, while the lowest passing rate was 49% in the 85-question examination. For ChatGPT, the highest passing rate was 14% in the 25-question examination, after which the passing rate continuously dropped as the questions increased, reaching 4% in the 85-question examination.

**Figure 2:**
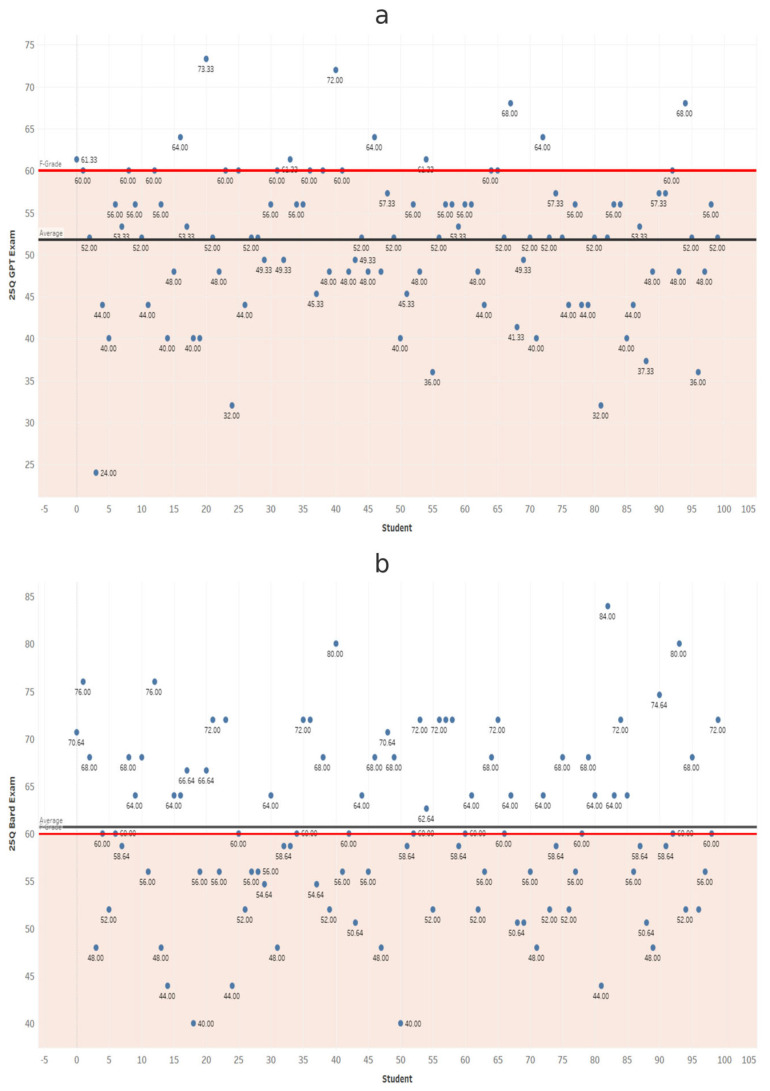
Performance of ChatGPT and Gemini (formerly Bard) on the 25-question examination. Mean percentage scores for (**a**) ChatGPT and (**b**) Gemini across 100 simulated examinations using 25 MCQs.

**Figure 3:**
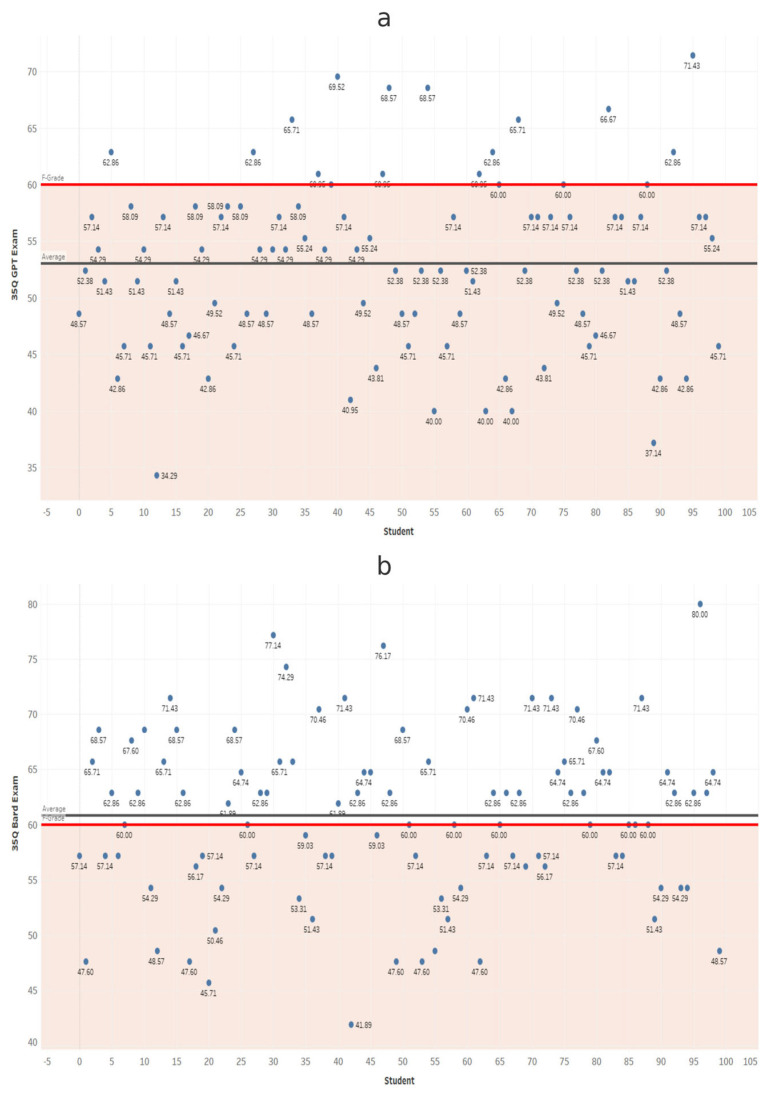
Performance of ChatGPT and Gemini (formerly Bard) on the 35-question examination. Mean percentage scores for (**a**) ChatGPT and (**b**) Gemini across 100 simulated examinations using 35 MCQs.

**Figure 4:**
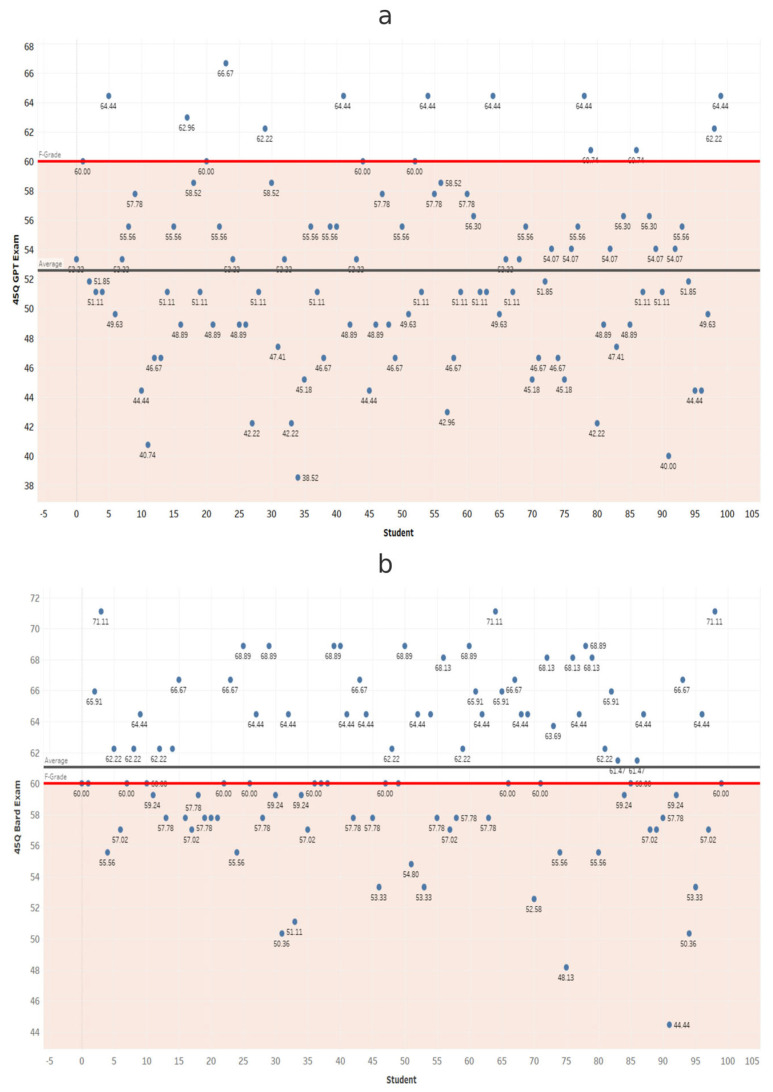
Performance of ChatGPT and Gemini (formerly Bard) on the 45-question examination. Mean percentage scores for (**a**) ChatGPT and (**b**) Gemini across 100 simulated examinations using 45 MCQs.

**Figure 5:**
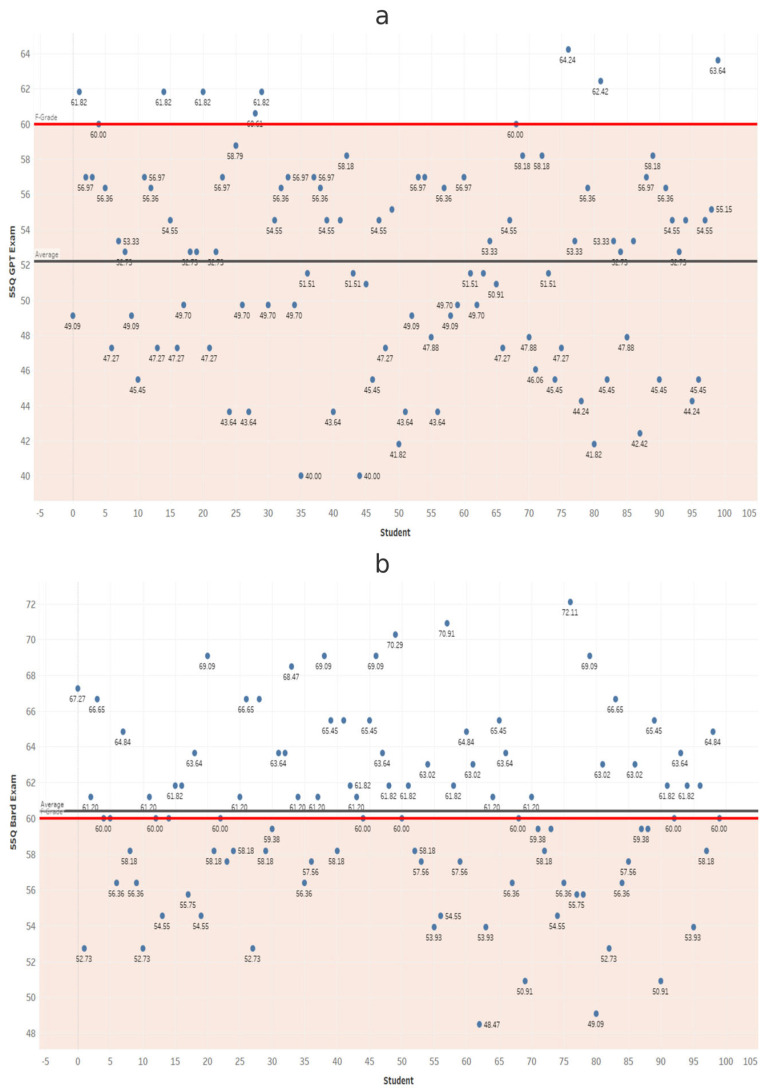
Performance of ChatGPT and Gemini (formerly Bard) on the 55-question examination. Mean percentage scores and standard deviations for (**a**) ChatGPT and (**b**) Gemini across 100 simulated examinations using 55 MCQs.

**Figure 6:**
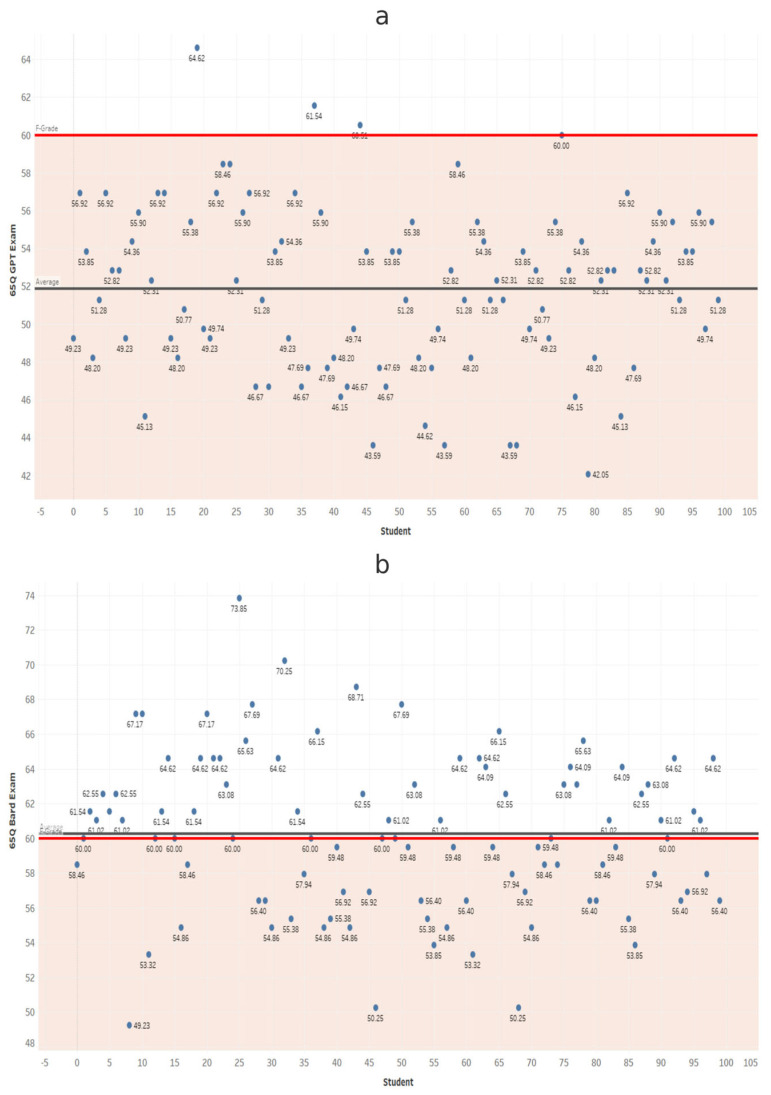
Performance of ChatGPT and Gemini (formerly Bard) on the 65-question examination. Mean percentage scores and standard deviations for (**a**) ChatGPT and (**b**) Gemini across 100 simulated examinations using 65 MCQs.

**Figure 7:**
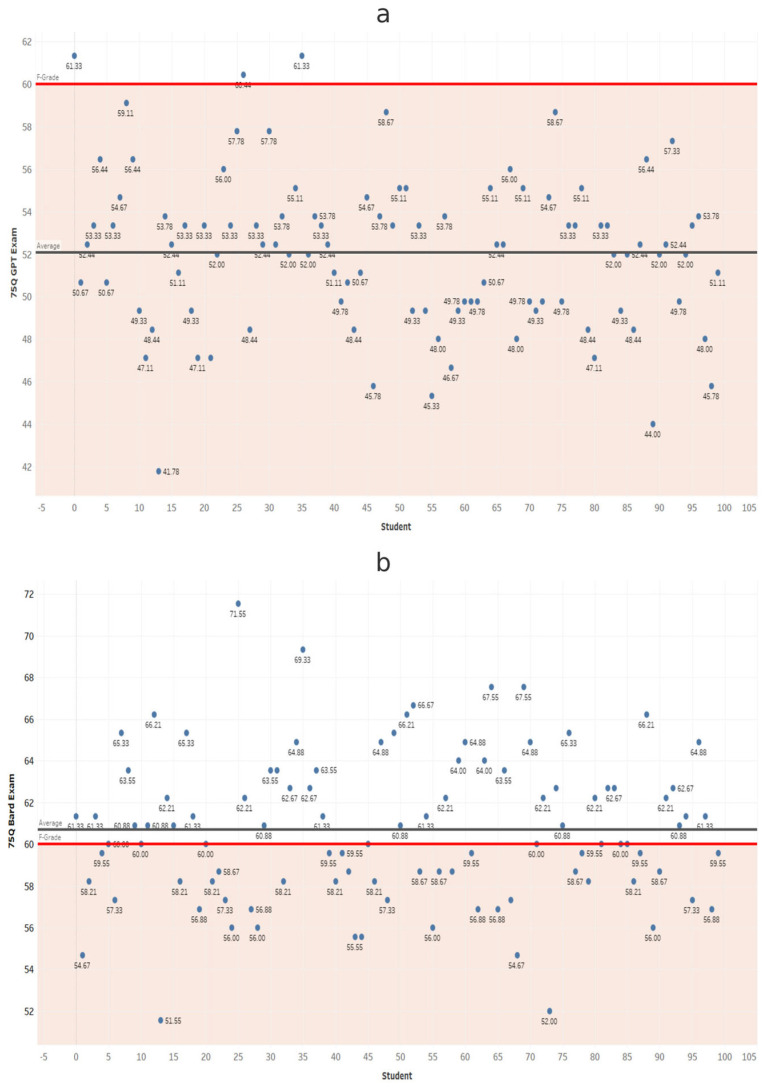
Performance of ChatGPT and Gemini (formerly Bard) on the 75-question examination. Mean percentage scores and standard deviations for (**a**) ChatGPT and (**b**) Gemini across 100 simulated examinations using 75 MCQs.

**Figure 8:**
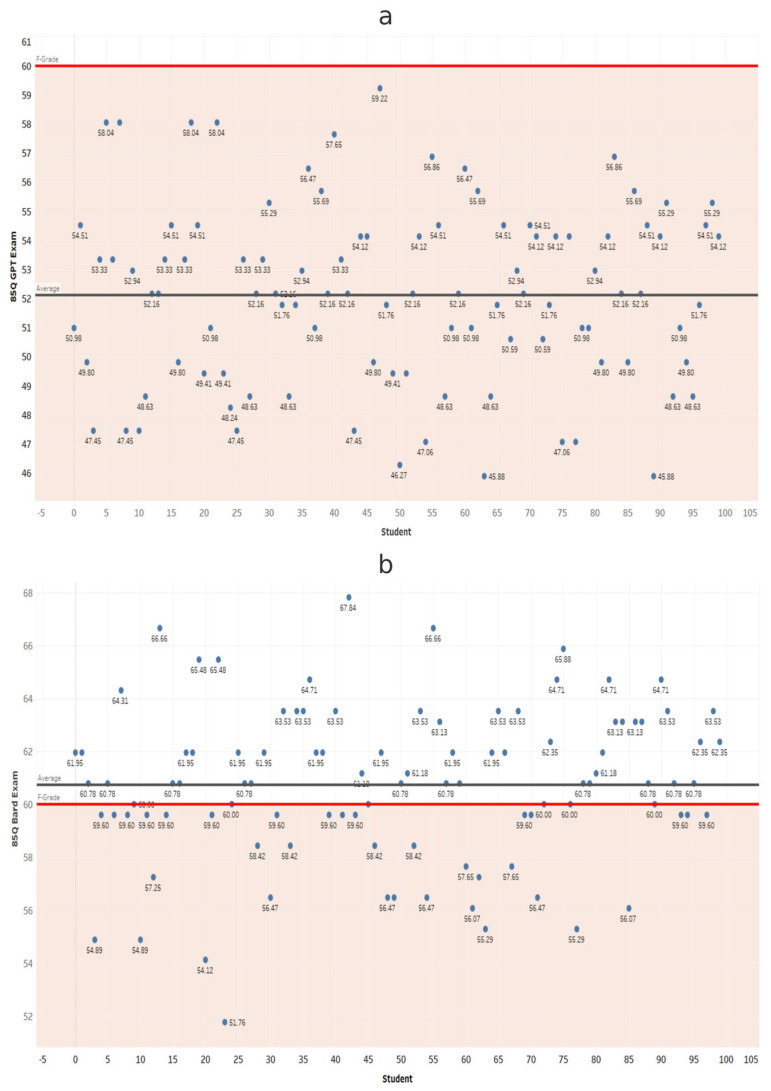
Performance of ChatGPT and Gemini (formerly Bard) on the 85-question examination. Mean percentage scores and standard deviations for (**a**) ChatGPT and (**b**) Gemini across 100 simulated examinations using 85 MCQs.

**Figure 9:**
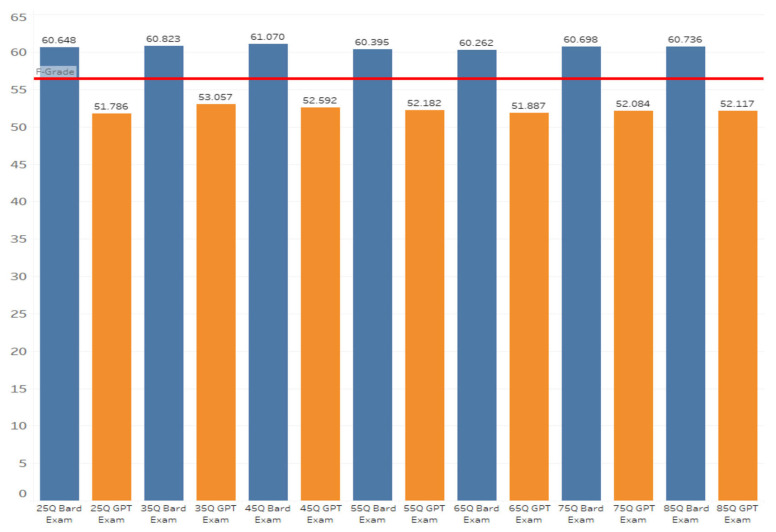
Comparison of overall mean scores (Y axis) across all examination lengths (X axis). Aggregated comparison of ChatGPT and Gemini (formerly Bard) mean scores across the seven examination lengths (25–85 questions), illustrating the consistent performance gap between LLMs.

At each exam length, scores from ChatGPT and Gemini were compared using independent-samples *t*-tests (Table 1). Gemini scored significantly higher in all comparisons (*p* < 0.001). These results confirm that Gemini significantly outperformed ChatGPT at every exam length (Figure 2, Figure 3, Figure 4, Figure 5, Figure 6, Figure 7, Figure 8 and Figure 9).

One-way ANOVA was used to assess whether examination length had a significant effect on scores within each LLM. In ChatGPT (Table 3), a statistically significant difference in mean scores was found across the seven exam lengths: F(6, 693) > 3.5, *p* < 0.01. Post hoc comparisons showed small but statistically significant fluctuations due to random sampling, although ChatGPT’s overall performance remained consistently low. In Gemini (Table 4), similarly, examination length significantly affected Gemini scores: F(6, 693) > 2.9, *p* < 0.05. However, post hoc comparisons demonstrated that Gemini’s performance showed minimal practical variation (≈1.0% difference across exam lengths), indicating robustness to question count.

A two-way ANOVA tested the effects of LLM Type (ChatGPT vs. Gemini), Question Count (25–85 questions), and the interaction term. LLM type was significant: F(1, 1386) = 118.05, *p* < 0.001 (Table 5). Gemini scores were consistently higher than ChatGPT scores. Question Count was significant: F(6, 1386) = 4.23, *p* = 0.0003. Longer exams produced slightly lower mean scores for both models. The interaction between both factors (LLM × Question Count) was not significant: F(6, 1386) = 1.18, *p* = 0.31. This indicates that changes in exam length affected both LLMs similarly; Gemini did not gain or lose a relative advantage with longer tests. Across all analyses, Gemini consistently demonstrated higher mean scores, higher passing rates, lower variance, greater robustness to exam length, and greater stability under random sampling. ChatGPT’s performance remained below passing thresholds in all conditions.

## 4. Discussion

To the best of our knowledge, this is the first study to assess ChatGPT and Gemini responses to MCQs about dental caries and how this might affect student performance. As cutting-edge AI language models, ChatGPT and Gemini were designed to generate natural-sounding responses during conversations. They achieve this using self-attention mechanisms and leveraging a vast amount of training data. One of their strengths is the ability to handle complex dependencies and to produce contextually appropriate, coherent responses.

This study compared the performance of two widely used large language models (LLMs), ChatGPT and Google Gemini, on a set of validated, domain-specific dental caries multiple-choice questions (MCQs) using a simulated student examination framework. Across all seven examination lengths, Gemini consistently achieved significantly higher accuracy and passing rates than ChatGPT. These findings provide important insight into the current capabilities and limitations of LLMs within dental education and highlight the need for cautious integration of these tools in knowledge assessment.

Gemini’s superior performance aligns with emerging evidence in medical and biomedical evaluation tasks, suggesting that Gemini’s underlying architecture may provide improved factual recall, reduced hallucination frequency, and more stable reasoning pathways compared with earlier LLMs. While ChatGPT also demonstrates strong linguistic capabilities, its accuracy in specialized biomedical topics remains variable. Prior research has shown that performance disparities often become more pronounced when LLMs are presented with granular, domain-specific content such as microbiology, pathology, and dental cariology fields, where factual precision is essential for clinical decision-making [45].

The consistency of Gemini’s performance across different examination lengths indicates that its accuracy is relatively insensitive to the size of the question pool. In contrast, ChatGPT exhibited more fluctuation across exam lengths, likely reflecting increased sensitivity to question sampling variability. These differences suggest that Gemini’s training data and model tuning may better support structured recall in low-context, high-precision testing environments. The two-way ANOVA results further support this interpretation, demonstrating a strong main effect for LLM type but no interaction between LLM type and exam length. This means that the advantage of Gemini over ChatGPT is stable regardless of the number of questions provided [21,46,47].

The null hypothesis was rejected. Gemini had significantly higher passing rates than ChatGPT in all seven examination groups. This could be attributed to the better training of Gemini than ChatGPT or the unavailability of sufficient information related to dental caries in both domains, particularly ChatGPT. It was also noticed that as the number of questions in the tests increased, Gemini obtained higher passing rates than ChatGPT. This could be related to the ability of the Gemini platform to train itself in the previous questions and to perform better in the following questions.

Several important pedagogical implications arise from these results. First, although Gemini outperformed ChatGPT, neither LLM achieved consistently high mastery across all caries-related content. This reinforces existing concerns that LLMs may provide superficially plausible but occasionally incorrect information, which could mislead learners who rely on these tools without appropriate supervision or prior knowledge. In the context of dental education, where foundational content informs clinical decisions, inaccurate LLM-generated responses may compromise student understanding and competency development [17]. Second, the use of LLMs as automated assessment tools remains premature. Despite improvements in accuracy, Gemini’s performance does not meet the reliability standards expected for summative assessment or for generating validated examination items. Educators should therefore refrain from replacing traditional evaluation methods with LLM-based systems. Instead, LLMs may offer value as supplementary tools for formative, low-stakes learning activities, such as generating study prompts, summarizing foundational concepts, or providing early-stage feedback [45]. However, the limitations demonstrated in this study suggest that human oversight remains essential. Third, the results contribute to broader discussions regarding the role of AI in competency-based dental education. As curricula increasingly incorporate technology-enhanced learning, LLMs may serve as adjunctive educational resources that promote self-directed learning. Yet, content validation, accuracy checks, and cross-referencing with evidence-based guidelines must be emphasized to prevent the propagation of misinformation. Faculty development programs may be beneficial to help educators understand both the strengths and constraints of AI systems when integrating them into instructional design [15,16,18,20,28,45,47]. This study also underscores key challenges inherent to LLM evaluation. For instance, LLMs are non-deterministic systems whose responses may vary across queries and over time due to model updates. Additionally, their accuracy on MCQs does not necessarily translate to diagnostic reasoning or clinical judgment, where contextual interpretation and multifactorial decision-making are required. Further research is necessary to determine whether LLMs can support higher-order cognitive processes relevant to dental training. Overall, the findings of this study reinforce the emerging perspective that LLMs can offer supportive educational benefits but should not yet be considered reliable sources of validated knowledge or independent assessment tools in dentistry [45]. Gemini’s better performance suggests an improved ability to process factual recall tasks, but both LLMs require substantial domain-informed constraints to ensure safe and accurate deployment in educational environments.

For practical use, this finding suggests that the stability of LLM-generated answers can depend on how examination questions are sampled. In educational settings where LLMs may be used as study aids, answer generators, or components of intelligent tutoring systems, instructors and developers should be aware that some models are more sensitive to variations in question composition than others. This sensitivity implies that the reliability of LLM-assisted performance may fluctuate across different exam versions, highlighting the importance of validating model behavior across multiple randomized test sets rather than relying on a single fixed examination.

This study highlights several limitations in evaluating LLMs, specifically focusing on simulated students in dental education. Firstly, while 1400 simulated examinations create a robust dataset, they cannot fully capture the variability of actual learners. The research is confined to dental caries, limiting generalizability across various dental disciplines. Additionally, utilizing a static question bank restricts the diversity of assessment items. As LLMs are non-deterministic and subject to continuous updates, performance measures could change over time with versions like ChatGPT or Gemini. Moreover, standardization of prompts does not eliminate sensitivity to structure, affecting accuracy. The study also primarily assessed answer accuracy without evaluating the quality of explanations, a key educational factor. Finally, the absence of real students prevents exploration of human–AI hybrid learning outcomes. Future research should aim to integrate LLMs in real instructional environments and examine their long-term educational effects.

However, the use of LLMs in dental education has various ethical considerations. Privacy protection is paramount, as it ensures secure storage and restricted access to data collected through LLM-powered tools and systems [48,49]. LLMs should complement rather than replace teachers, respecting the essential roles of human instructors in providing guidance and mentorship [50]. Careful design and implementation are necessary to prevent bias perpetuation within LLM algorithms [51]. Autonomy should be upheld to allow students to make their own choices and decisions, with LLMs serving as supportive tools [52]. Transparency is critical to ensure that students and instructors are informed about data collection, usage, and protection [53]. Accountability is also essential for establishing explicit standards and guidelines for LLM-powered tools and for holding individuals and organizations responsible for ethical conduct [54,55]. Considering these ethical considerations, LLMs can be used to support students and to promote ethical and effective dental education. The significant opportunity for AI integration in educational settings is significant, as it can enhance student engagement and learning outcomes. AI platforms can provide personalized feedback, assess student performance, and automate administrative tasks. AI systems can collect and analyze vast volumes of data, providing valuable insights for educational strategies. Continuous monitoring and updating of AI algorithms are necessary for equitable education.

The existing research on the performance of LLMs in dental question answering remains unexplored, and our work addresses this gap. Our study provides insights into the application of LLMs in dental education. On one hand, LLMs have the potential to assist students in learning dental knowledge, such as serving as virtual tutors to guide students. On the other hand, educators should carefully consider preventing students from using LLMs to cheat in dental exams or assignments.

## 5. Future Directions

Future investigations should compare a broader range of examination materials from various dental disciplines, adapting methodologies to the unique needs of dental students and evaluating their learning results. Research should include a wider array of confounding factors, enhancing understanding of AI’s applications in dental education. Studies must expand evaluation of large language models (LLMs) beyond dental caries to encompass restorative dentistry, endodontics, and more, assessing higher-order cognitive skills through case-based questions. Continuous monitoring of LLM performance is essential due to the evolving nature of these models. Future studies should involve human participants to evaluate usability and learning impacts while exploring hybrid human–AI educational frameworks to balance efficiency and safety. Finally, research should focus on reducing hallucinations, improving prompt designs, and enhancing domain-specific fine-tuning for LLMs in professional education. Despite this consistent advantage, neither LLM achieved performance levels sufficient for high-stakes assessment or autonomous knowledge validation in dental education. Gemini’s improved accuracy suggests potential value as a supplementary learning tool, but both models require careful oversight due to occasional incorrect responses and a lack of domain-specific reasoning. As LLMs continue to evolve, dental educators should approach their integration thoughtfully, leveraging their strengths for formative learning, content generation, or preliminary concept reinforcement while maintaining rigorous human control over assessment, curriculum design, and clinical instruction. Ongoing evaluation of LLM performance across broader dental domains and more complex scenarios is essential before these systems can be considered reliable educational instruments.

## 6. Conclusions

Within the limitations of our study, this study provides a comprehensive comparison of ChatGPT and Google Gemini in answering validated dental caries multiple-choice questions using a simulated examination model. Across all seven examination lengths, Gemini consistently outperformed ChatGPT in mean score, passing rate, and score stability. The statistical analyses confirmed significant main effects for both LLM type and question count, with Gemini demonstrating superior accuracy independent of exam length.

## Figures and Tables

**Table 1:** Comparison of mean examination scores and standard deviations for ChatGPT and Gemini across all examination lengths.

Examination Length	N per LLM	ChatGPT Mean (%) ± SD	Gemini Mean (%) ± SD	Difference (%)	*t*-Test (*p*-Value)
25 questions	100	53.2 ± 6.4	61.6 ± 5.8	+8.4	<0.001
35 questions	100	52.4 ± 5.9	61.3 ± 5.4	+8.9	<0.001
45 questions	100	52.1 ± 6.1	60.9 ± 5.2	+8.8	<0.001
55 questions	100	51.7 ± 5.8	60.7 ± 5.1	+9.0	<0.001
65 questions	100	51.5 ± 5.5	60.6 ± 4.9	+9.1	<0.001
75 questions	100	51.2 ± 5.6	60.5 ± 5.0	+9.3	<0.001
85 questions	100	51.0 ± 5.7	60.4 ± 4.8	+9.4	<0.001

Values represent mean percentage scores (correct answers ÷ total questions) ± standard deviation. Statistical test: Independent-sample *t*-tests for each exam length (all *p* < 0.001). All comparisons show significantly higher scores for Gemini (*p* < 0.001).

**Table 2:** Passing rates (≥60%) for ChatGPT and Gemini by examination length.

Examination Length	N per LLM	ChatGPT Passing Rate (%)	Gemini Passing Rate (%)
25 questions	100	14%	59%
35 questions	100	11%	57%
45 questions	100	10%	55%
55 questions	100	8%	52%
65 questions	100	6%	51%
75 questions	100	5%	50%
85 questions	100	4%	49%

Passing rate, defined as achieving ≥60% correct answers. Statistical test: chi-square test of proportions (all *p* < 0.001).

**Table 3:** One-way ANOVA results for ChatGPT across examination lengths (25–85 questions).

Source of Variation	SS	df	MS	F	*p*-Value
Between groups	215.40	6	35.90	3.67	0.0014
Within groups	6775.72	693	9.78	—	—
Total	6991.12	699	—	—	—

One-way ANOVA evaluating the effect of test length on ChatGPT scores.

**Table 4:** One-way ANOVA results for Gemini across examination lengths (25–85 questions).

Source of Variation	SS	df	MS	F	*p*-Value
Between groups	178.92	6	29.82	2.94	0.008
Within groups	7027.31	693	10.14	—	—
Total	7206.23	699	—	—	—

One-way ANOVA evaluating the effect of test length on Gemini scores.

**Table 5:** Two-way ANOVA results (LLM × Question Count). Factors: (1) LLM type (ChatGPT vs. Gemini), (2) exam length (25–85 questions). N = 1400 simulated examinations. ns is non-significant.

Source	SS	df	MS	F Value	*p*-Value
LLM type	1452.80	1	1452.80	118.05	<0.001
Question count	312.42	6	52.07	4.23	0.0003
Interaction	87.36	6	14.56	1.18	0.31 (ns)
Residual	17,053.52	1386	12.30	—	—
Total	18,806.10	1399	—	—	—

Two-way ANOVA examining the main effects of LLM type and exam length and their interaction.

## Data Availability

The original contributions presented in this study are included in the article. Further inquiries can be directed to the corresponding author.
